# Lesion Volume Quantification Using Two Convolutional Neural Networks in MRIs of Multiple Sclerosis Patients

**DOI:** 10.3390/diagnostics12020230

**Published:** 2022-01-18

**Authors:** Marcela de Oliveira, Marina Piacenti-Silva, Fernando Coronetti Gomes da Rocha, Jorge Manuel Santos, Jaime dos Santos Cardoso, Paulo Noronha Lisboa-Filho

**Affiliations:** 1 Department of Physics, School of Sciences, São Paulo State University (UNESP), Bauru 17033-3600, SP, Brazil; marina.piacenti@unesp.br (M.P.-S.); paulo.lisboa@unesp.br (P.N.L.-F.); 2 Department of Neurology, Psychology and Psychiatry, Medical School, São Paulo State University, Botucatu 18618-687, SP, Brazil; fcoronetti@mac.com; 3 Department of Mathematics of School of Engineering, Polytechnic of Porto (ISEP), 4249-015 Porto, Portugal; jms@isep.ipp.pt; 4 Institute for Systems and Computer Engineering, Technology and Science (INESC TEC) and Faculty of Engineering, University of Porto, 4200-465 Porto, Portugal; jaime.cardoso@inesctec.pt

**Keywords:** multiple sclerosis, MRI, machine learning, brain extraction, lesion volume quantification

## Abstract

Background: Multiple sclerosis (MS) is a neurologic disease of the central nervous system which affects almost three million people worldwide. MS is characterized by a demyelination process that leads to brain lesions, allowing these affected areas to be visualized with magnetic resonance imaging (MRI). Deep learning techniques, especially computational algorithms based on convolutional neural networks (CNNs), have become a frequently used algorithm that performs feature self-learning and enables segmentation of structures in the image useful for quantitative analysis of MRIs, including quantitative analysis of MS. To obtain quantitative information about lesion volume, it is important to perform proper image preprocessing and accurate segmentation. Therefore, we propose a method for volumetric quantification of lesions on MRIs of MS patients using automatic segmentation of the brain and lesions by two CNNs. Methods: We used CNNs at two different moments: the first to perform brain extraction, and the second for lesion segmentation. This study includes four independent MRI datasets: one for training the brain segmentation models, two for training the lesion segmentation model, and one for testing. Results: The proposed brain detection architecture using binary cross-entropy as the loss function achieved a 0.9786 Dice coefficient, 0.9969 accuracy, 0.9851 precision, 0.9851 sensitivity, and 0.9985 specificity. In the second proposed framework for brain lesion segmentation, we obtained a 0.8893 Dice coefficient, 0.9996 accuracy, 0.9376 precision, 0.8609 sensitivity, and 0.9999 specificity. After quantifying the lesion volume of all patients from the test group using our proposed method, we obtained a mean value of 17,582 mm^3^. Conclusions: We concluded that the proposed algorithm achieved accurate lesion detection and segmentation with reproducibility corresponding to state-of-the-art software tools and manual segmentation. We believe that this quantification method can add value to treatment monitoring and routine clinical evaluation of MS patients.

## 1. Introduction

Multiple sclerosis (MS) is known as one of the neurodegenerative diseases that affects the central nervous system [1]. Regions of demyelination, inflammatory processes, and axonal loss are characteristics of this disease, causing brain injuries that occur with higher incidence in white matter (WM) [2,3]. Magnetic resonance imaging (MRI) is the main imaging technology used to detect alterations in subjects with MS [4]. This imaging modality is an important part of the initial diagnosis and treatment of the illness, and it also provides essential information for monitoring the severity and activity of the disease in individuals with defined MS [4]. Moreover, radiological abnormalities can be detected even without the observation of clinical symptoms of the illness, making MRI analysis attractive for a premature diagnosis and anticipating the start of MS treatment [5].

Conventional MRIs, including T2-weighted (T2-w), T1-weighted (T1-w), T1-weighted contrast, and fluid attenuated inversion recovery (FLAIR) modalities, are applied to visualize and analyze overt lesions and assess brain atrophy in MS [4,6]. The visualization and evaluation of these lesions can provide quantitative information on inflammatory activity, as well as point to future brain atrophy and clinical incapacity due to the disease [7,8]. Furthermore, specifically considering MS, clinical assessments for monitoring its progression and evaluating the effectiveness of disease-modifying therapies and rehabilitation therapies can be performed with the quantitative definition of lesion load and volumetric analyses of the brain [9]. Manifestations of MS lesions are conspicuous on MRI, such as hyperintensities on FLAIR and T2-w modalities, and hypointensities on T1-w [4]. Advances in technology have improved the quality of images and the visual assessment of the lesions caused by multiple sclerosis, aiding medical diagnosis. Nevertheless, there is a need to improve methods, facilitating their applicability and validation, allowing these available tools to have a clinically appropriate use. Currently, the analysis and manual segmentation of MS lesions performed by a specialist is considered the gold standard in the evaluation of MRIs. However, this task presents a certain subjectivity with inter- and intra-observer variations, consuming time and depending on the degree of observer experience. Thus, a large number of computer programs for automatic detection and segmentation of MS lesion have been proposed in the literature [1].

These automated methods can be applied to analyze medical images with large datasets, including MRI, in order to help in the recognition and extraction of information in less time with good accuracy. The main challenges for automated programs reside in the fact that brain anatomy and lesion pathology present great biological variability, and guarantee robustness to the imperfections that can be generated during the process of MRI acquisition [10]. A variety of computational techniques, especially machine learning techniques, are used for automated analysis of MRIs of MS patients, with reviews accessible that analyze and assess the usefulness of these methods [2,10,11,12,13,14]. Deep learning is a subset of machine learning in which data-driven predictions from large datasets are performed by computer systems [15]. DL techniques, especially computational algorithms based on convolutional neural networks (CNNs), have become frequently used algorithms to perform feature self-learning, enabling segmentation of structures in the image useful for quantitative analysis of MRIs, including quantitative analysis of MS [14,16]. Most studies using CNN methods on images of patients with MS focus only on lesion segmentation. In addition to lesion segmentation, brain extraction is also a critical and important preprocessing step in MRI evaluation that can affect the accuracy of subsequent analyses [17,18]. Usually, image preprocessing in MS cases, can be performed manually with external software (FreeSurfer, SwissSkullStripper, Robust Brain Extraction-ROBEX, and Brain Extraction Tool-BET) [19,20,21,22,23]. Authors mention that such a process was previously performed without much detail. However, this function is dependent on external software and sometimes needs program-specific input parameters (depth, kernel size, iterations, and stiffness), that depends on specific prior knowledge. Furthermore, in this external software, there is a need to use a function to perform brain extraction and another additional function to perform segmentation and detection of possible lesions. Therefore, in the current study, we performed volumetric quantification of white matter lesions on MRIs of patients with MS by performing automatic brain and lesion segmentation using two CNNs. The first CNN was responsible for performing brain extraction, and the second CNN was responsible for lesion segmentation. Thus, our method performed both brain extraction and lesion detection functions at the same time, enabling volumetric quantification of the lesion.

## 2. Materials and Methods

### 2.1. Subject Sample and Image Data

This study included four datasets: one used for brain segmentation model training, two used for brain lesion segmentation model training, and one used for model testing. The first dataset included a training set from the Neurofeedback Skull-stripped (NFBS) repository, which contains a training subset of 80 T1-w MRI scans that were manually skull-stripped (brain mask) [24,25]. The second and third datasets included training sets for brain lesion detection and are publicly available: the second dataset was from the IBSI 2015 challenge, containing a training set of 21 scans (FLAIR modality) from five subjects [11]; and the third dataset was from MICCAI 2016, with MRI scans from 15 patients also acquired in FLAIR modality [26]. Both training sets also included the identification and manual lesion segmentation performed by a specialist. Finally, the fourth dataset was the test group, which was not public and included 50 individuals with MS (MRI modalities T1-w and FLAIR). Individuals in this last group (test) were diagnosed with multiple sclerosis according to the McDonald criteria [27,28] and were obtained from the Hospital of Clinics—Botucatu Medical School, Brazil (HC-FMB). All MRIs and diagnostic information of the patients were collected retrospectively between 2013 and 2019. MRIs from the test group were collected and used in compliance with the ethics committees of the authors’ institutions, and all patients provided written informed consent to use the images in this study. All datasets were fully anonymized for dissemination purposes. The image acquisition parameters and demographic information of subjects are detailed in Table 1.

### 2.2. Image Preprocessing

MRIs from training groups (NFBS, MICCAI 2016 and IBSI 2015) were already preprocessed, and test group MRIs were preprocessed with the same six steps: (1) resliced to 1 mm^3^; (2) anisotropically diffused; (3) rigidly registered; (4) normalized and standardized; (5) skull-stripped (brain extraction); and (6) intensity-corrected due to inhomogeneity of the magnetic field. Initially (first step), the T1-w and FLAIR images of the individuals in the test group were resliced at isotropic resolution using cubic spline interpolation to the axial 1 mm^3^. In the second step, to reduce possible noise in the image, an anisotropic diffusion filter was applied. The diffusion coefficient was chosen to vary spatially to promote smoothing within the region rather than between regions [29,30]. Next, the FLAIR slices were aligned with the T1-w slices using rigid-body registration. We aligned the first slice of the FLAIR sequence with the first image of the T1-W and the second slice of the one sequence with the second slice of another modality up to the nth slice of the entire modality. In the fourth step, the image intensities were normalized. This normalization of a particular slice of the modality was performed by subtracting the mean value of that image and dividing by its standard deviation. In the fifth step, the brain extraction (skull-stripping process) was performed using the CNN training model with the dataset from NFBS.

**Figure 1:**
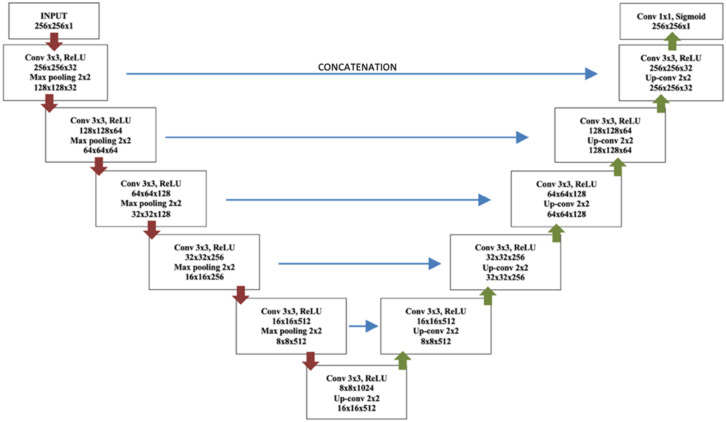
U-Net architecture for brain extraction.

Figure 1 shows the complete network architecture for brain segmentation. The CNN framework for brain extraction is a U-Net architecture and consists of convolutional layers, 5 Max pooling layers, 5 Up-sampling layers, and a sigmoid layer at the end. The left side of the architecture in Figure 1 represents an encoder, and the right side—a symmetric decoder.

Most brain extraction methods have been produced for T1-w, since this is one of the most common modalities in neurological imaging, providing different signal intensity to different brain tissues [31]; hence, this modality was used as the input image. Each block in the encoder was responsible for feature extraction with a convolutional layer, batch normalization, a rectified linear unit (ReLU), and Max pooling [32,33]. Each block in the decoder consisted of Up-sampling layers that provided precise localization instead of Max pooling layers. Thus, the CNN layers learned the transformations from the intensities to the feature maps to obtain the final probabilistic brain mask (brain extraction). The output layer was a convolutional layer followed by a sigmoidal activation function to identify whether brain was present or not. The training model was applied to the T1-w modalities of the test group as input and the brain mask was obtained as output. Then, the brain mask from T1-w was used as model for skull-stripping on FLAIR images, and we obtained the brain extraction in both modalities of the test group. Finally, to improve the image homogeneity, in the sixth step of preprocessing, we performed image bias correction after brain stripping [34].

### 2.3. Brain Lesion Segmentation

**Figure 2:**
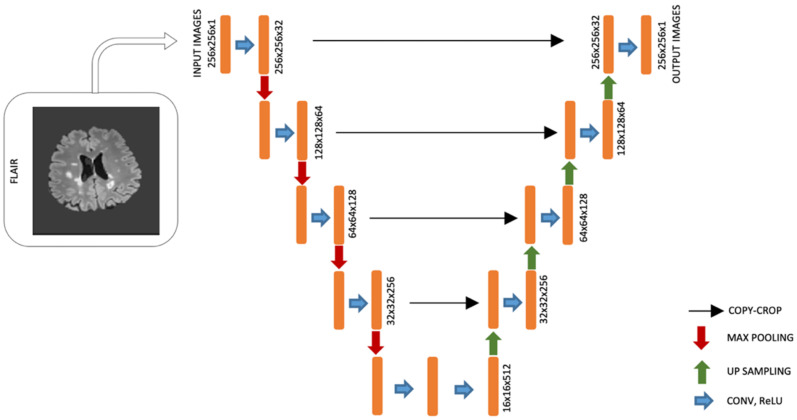
U-Net architecture for lesion detection.

A different CNN model was used to perform brain lesion segmentation using datasets from the IBSI 2015 Challenge and MICCAI 2016 as input images from the network. The U-Net network architecture (adapted from Ghosal et al. [35]) contains a contraction path and another symmetric expansion path used to record context and precise localization, respectively, see Figure 2. The new McDonald guidelines emphasize the importance of lesion location rather than the number of lesions, and white lesions are best visualized with FLAIR sequences [36]; therefore, this modality was used as the input image. For the convolution process in the down-layer and deconvolution process in the up-layer, kernels (3 × 3) were used. ReLU was used as the activation function. A 2 × 2 Max pooling operation (stride 2) was applied to reduce the image size in the down-layer. We used the concatenations with the respective cropped feature map from the down-layer into the corresponding input of the convolutional layer in the up-layer. The output layer was a convolutional layer that detects whether a brain lesion is or is not present.

Both CNNs were trained with a batch size value of 32, the Adam optimizer, and a learning rate of 0.001. Cross-entropy was applied as the loss function, and we trained the models for 60 epochs. To test both models, we employed a cross-validation strategy of 80:20 split (10 times). The Dice coefficient, accuracy, sensitivity, precision, and specificity were used as performance measures to evaluate the final models. We implemented the CNN framework in Python with deep learning tools, TensorFlow and Keras.

### 2.4. Brain Lesion Identification and Quantification from Test Group

The trained models were applied to the MRIs (previously preprocessed) of the test group to obtain brain lesion segmentation. Shortly thereafter, memberships and a binary mask (after detection by thresholding the probabilistic memberships) were generated for each subject. Lesion volume quantification was obtained with a count of the segmented voxels and presented in mm^3^. Finally, we compared the automatic volume quantification by the CNNs with the volume provided by manual annotations of an expert.

## 3. Results

**Figure 3:**
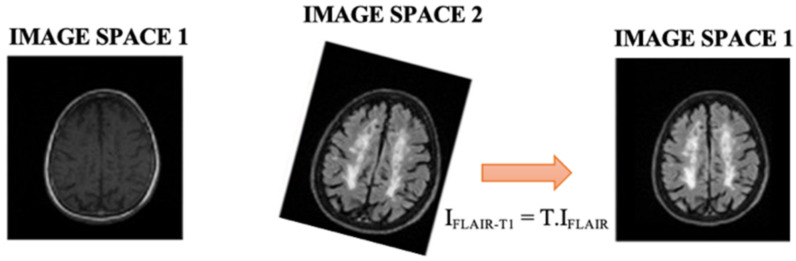
FLAIR volume spatially aligned to T1-w volume.

In this work, two automated methods for segmentation of brain (skull-stripper) and white matter lesions were applied to determine brain lesion volume in individuals with MS from a private test group. MRIs with T1-w and FLAIR modalities were used for the analysis, which were first preprocessed. The first step of the image preprocessing was to transform the original size images (0.43 × 0.43 × 4.5 mm^3^ to FLAIR and 0.47 × 0.47 × 4.5 mm^3^ to T1-w) to the axial 1 mm^3^. We then reduced the noise in the second step. Subsequently, the FLAIR slices were aligned with the T1-w slices. By applying the registration transform T to the FLAIR initial volume (I_FLAIR_), we generated a new volume spatially aligned with the T1-w volume (I_FLAIR-T1_), see Figure 3.

**Figure 4:**
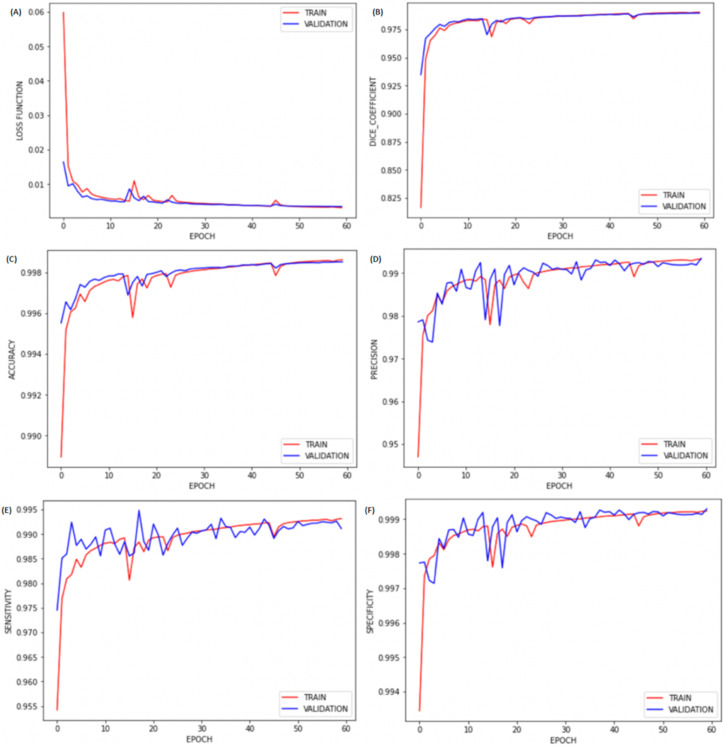
Plots of (**A**) loss, (**B**) Dice coefficient, (**C**) accuracy, (**D**) precision, (**E**) sensitivity, and (**F**) specificity by epochs for brain extraction.

After spatial alignment, we resized all images to 256 × 256 × j. Next, all these images were normalized between 0 and 1 to improve training. Then, we performed brain extraction (skull-stripping process) using the first CNN training model to perform automatic brain segmentation for all patients in the test group. The performance of the proposed brain segmentation (extraction) framework with binary cross-entropy as the loss function is reported in Table 2. The results, represented in Figure 4, show a correlation of sensitivity, specificity, accuracy, and Dice coefficient with the number of epochs for our brain segmentation. We found that the training with 60 epochs was sufficient, as no significant improvement was observed after 45 epochs. We also compared our brain segmentation method with other available software, and we found that the value of the Dice coefficient obtained was higher than those obtained by BET (0.8319) [19], FreeSurfer (0.9020) [22], and SwinissSkullStripper (0.9140) [21].

**Figure 5:**
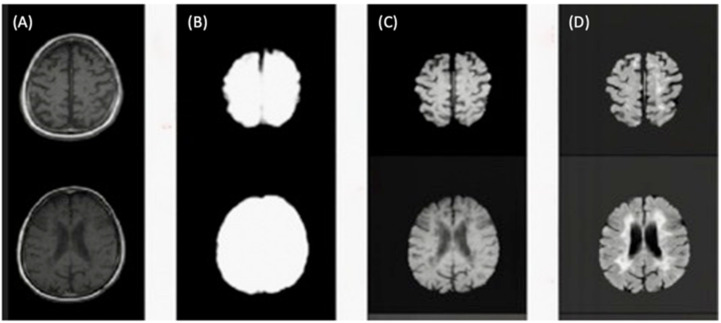
(**A**) Slices from original T1-w image. (**B**) Brain mask through skull-stripping using CNN. (**C**) Brain extraction in T1-w. (**D**) Brain extraction in FLAIR image.

Figure 5 illustrates the skull-stripping process using the U-Net architecture for brain extraction described in step five.

**Figure 6:**
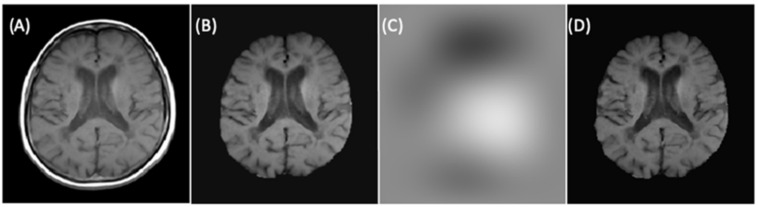
(**A**) Rigidly registered image. (**B**) Skull-stripped image. (**C**) Bias correction filter. (**D**) Final image.

**Figure 7:**
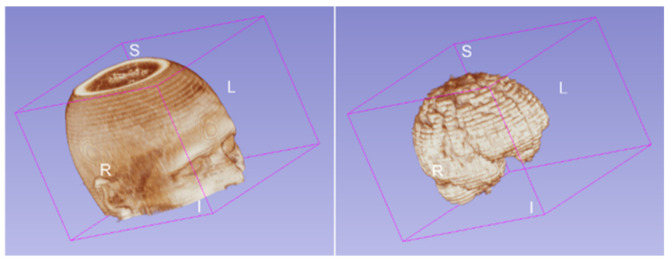
Brain volume after the brain extraction process applied to all slices for one patient.

Finally, to improve the signal intensity variability in MRIs caused by magnetic field inhomogeneities, we applied the image bias correction filter (N4ITK) in step six of the preprocessing after automatic brain extraction. Figure 6 illustrated an example of a slice with the application of the bias correction step. The brain extraction process and bias correction were applied to all the slices in the exam (each patient), and we were able to see the volumetric representation of the extracted brain (see Figure 7).

**Figure 8:**
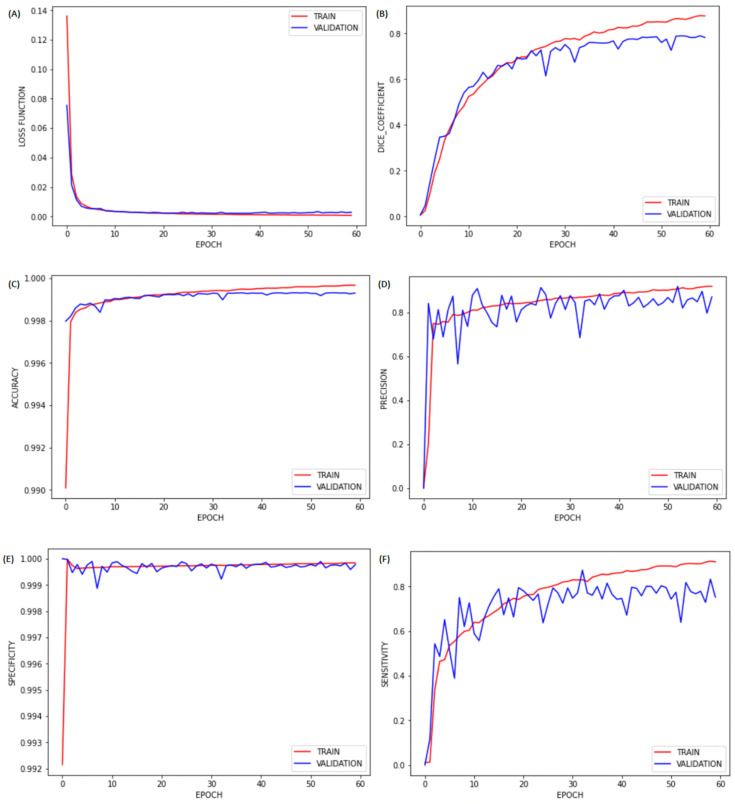
Plots of (**A**) loss, (**B**) Dice coefficient, (**C**) accuracy, (**D**) precision, (**E**) sensitivity, and (**F**) specificity by epochs for brain lesion detection.

After preprocessing the image, to detect MS lesions for each subject, we performed the second CNN training using the dataset from the IBSI 2015 and MICCAI 2016. In the proposed brain lesion segmentation/detection framework with binary cross-entropy as the loss function, we obtained the values shown in Table 2 (second CNN). The graphs in Figure 8 indicate that the training with 60 epochs was sufficient, as there was no significant improvement after about 50 epochs.

Table 3 presents the performance of our second CNN in comparison with that of other previous studies. As can be seen, our CNN had better results for Dice coefficient, precision, and specificity metrics, while also exhibiting excellent accuracy, which indicates that the proposed CNN could identify brain lesion volumes with higher precision and accuracy than other previous methods.

**Figure 9:**
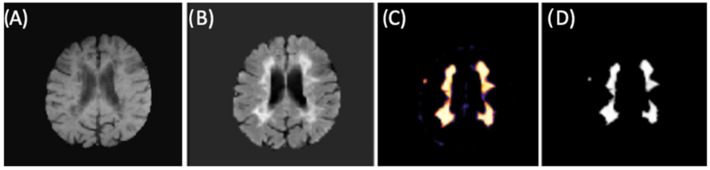
CNN example for lesion segmentation. (**A**) T1-w image after preprocessing and brain extraction. (**B**) FLAIR image after preprocessing and brain extraction (second CNN input). (**C**) Lesion memberships. (**D**) Binary mask by thresholding the probabilistic memberships.

The final training model obtained from the second CNN (as described in Materials and Methods 2.3 and in Figure 2) was used for brain lesion detection, where convolutional blocks were applied to the FLAIR input. From binary mask (outputs), we could detect lesions within new individuals (images) from our test group. Figure 9 shows an example of lesion segmentation on a patient from the test group.

**Figure 10:**
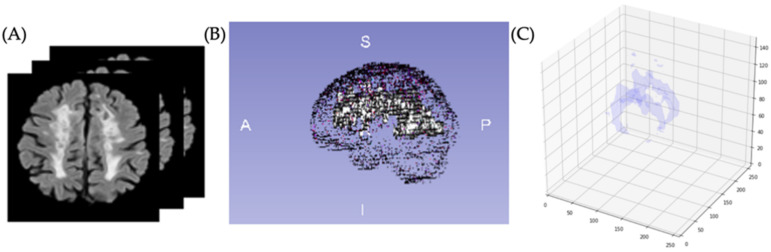
(**A**) Images for identification and segmentation of sclerotic lesions. (**B**) Brain with lesion volumetric representation. (**C**) Segmented volumetric lesion.

Because lesion volume definition is a relevant metric for assessing disease evolution and monitoring treatment, we determined volumetric quantification of the brain lesion by counting the detected voxels from the binary mask (CNN output). After volumetrically quantifying the lesions of all patients from the test group, we obtained a mean value of 17,582 mm^3^; Figure 10 shows an example where the segmentation was performed on all slices and volumetrically represented.

**Figure 11:**
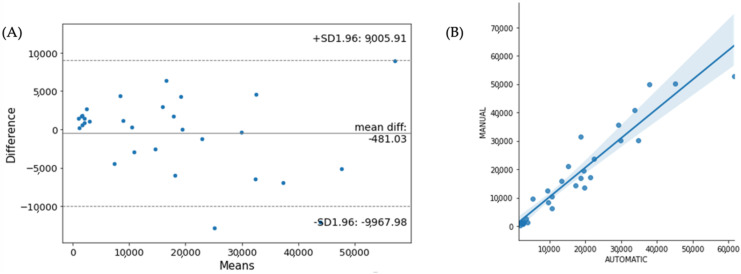
(**A**) Bland-Altman plot and (**B**) correlation for total lesion volume agreement between automatic and manual lesion detection.

In terms of reproducibility and validation of lesion segmentation, we compared the automated lesion volume (using the CNN in our method) with the expert-defined lesion volume by using a Bland–Altman plot (see Figure 11A). We found similar lesion volumes between the two lesion segmentation methods, showing a high degree of agreement with R^2^ = 0.95 (see Figure 11B).

## 4. Discussion

**Figure 12:**
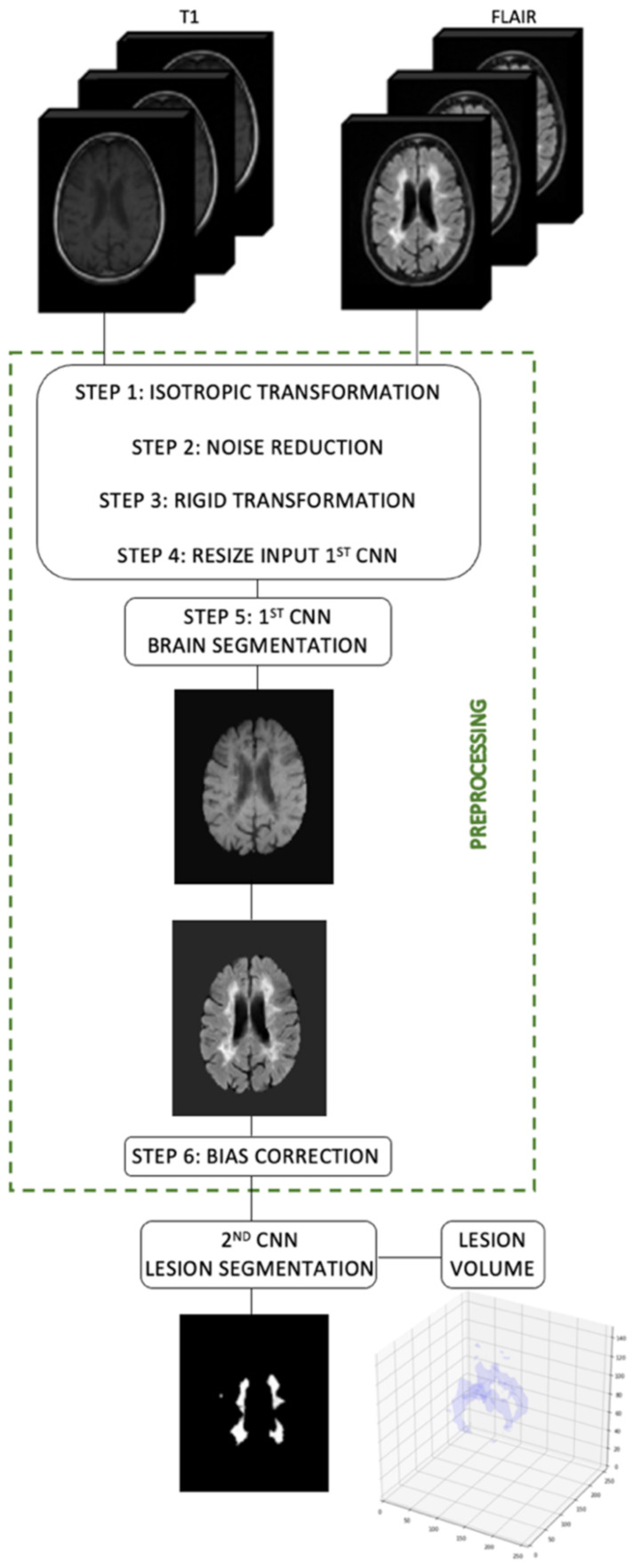
Flowchart of our proposed method using two CNNs for brain lesion volume quantification.

We proposed a method for volumetric lesion quantification using two CNNs in MRIs of MS patients. Our networks were applied in two different moments, first for brain segmentation (skull-stripping and brain extraction) and second for lesion segmentation. Figure 12 shows our proposed method under a general schematic view.

Image detection or segmentation is an essential step in several medical applications involving visualization, measurements, registration, and computer-aided diagnosis [38]. Accurate and robust segmentation of MRI injuries can serve as important information about disease status, progression, response to drugs [13]. The use of machine learning, specifically DL techniques, in healthcare continues to evolve. DL learns inherent imaging parameters and does not need extensive post-processing to eliminate false positives, as is usual in other MS detection methods [14]. In addition, a popular DL network architecture in medical image evaluation uses a CNN-based algorithm to perform detection and segmentation [14].

Brain extraction and segmentation of brain lesions are difficult but crucial tasks to evaluate on MRIs of individuals with neurodegenerative disorders, especially those with MS [15]. In recent years, numerous deep learning techniques have been applied for brain segmentation, but not specifically for images of MS subjects. In MRI of multiple sclerosis, these techniques have only been applied directly for lesion segmentation. Kamnitsas et al. [39] proposed an architecture with dual-pathway, 11-layers-deep, and three-dimensional CNN for the difficult task of targeting brain injuries using five sequences: Proton Density (PD), MPRAGE, axial T2 and FLAIR, and Gradient-Echo (GE). Roy et al. [13] applied a fully convolutional neural network model to target MS lesions, in which parallel pathways of convolutional filters were first applied to multiple contrast and then outputs were concatenated. After that, another group of convolutional filters was used on the joined output. Gabr et al. [14] used four different image sequences to developed a full CNN model to segment brain tissues in the T2-w modality. In another study, the authors performed MS lesion segmentation with a lightweight deep learning framework [35] using five different modalities. In the last year, we also performed lesion segmentation in MS subjects using CNN [15,18].

Regarding brain extraction (or brain segmentation, or skull-stripper), Hwang et al. [33] suggested the application of 3D U-Net for skull-stripping in brain MRIs from individuals with multiple subclinical and clinical psychiatric symptoms, but not including MS. Tao and Chang [40] adopted an automated skull-stripping method based on deformable surface models and fuzzy tissue classification without specifying the individual conditions. Moeskops et al. [41] proposed an algorithm for automatic segmentation of anatomical magnetic resonance brain images, but not particularly for MS patients, adopting multiple classes on a multiscale CNN. In another study, Eskildsen et al. [31] proposed a new algorithm based on nonlocal segmentation technique, aimed at producing brain extraction with precision and consistency. As mentioned in these and other studies, CNNs and DL-based algorithms have achieved excellent performance in targeting different structures and tissues in biomedical images, with an accuracy close to that of human performance [33]. However, most of these studies about brain lesion segmentation use software to perform brain extraction manually or a database with images already skull-stripped, applying DL only to lesion detection and with certain limitations. In addition, these studies for brain extraction do not mention MRIs from MS patients in particular. Thus, in this study, we decided to implement the first framework for research about brain extraction in MRI images of MS patients, considering that brain extraction is also a fundamental step in image preprocessing and the applicability of a DL technique will increase the accuracy in this process. Furthermore, since brains lesion volume is a relevant outcome measure for evaluating disease diagnosis and progression, we implemented the second framework for brain lesion segmentation to perform volumetric quantification of brain lesions using only two different basic modalities. Several studies use and depend on various image modalities (between three and five) to perform this task [2,14]. Although we validated using only one modality, other contrast sequences can be included in the framework. Moreover, we proposed the segmentation with one sequence but with high accuracy. Future work will include further longitudinal evaluation of the atrophy analysis and its possible association with load lesion.

## 5. Conclusions

In conclusion, we implemented a method to quantify lesions in MRIs of patients with multiple sclerosis using two CNNs for automatic segmentation (extraction) of the brain and lesions. The proposed method demonstrated accurate segmentation of lesions with reproducibility comparable to that of state-of-the-art software tools and manual segmentation. We believe that this quantification method can add value to treatment monitoring and routine clinical assessment of patients with MS.

## Figures and Tables

**Table 1:** Demographics data and acquisition details for the training and testing databases.

	Scanner Model and Site	Sequence	Voxel Size (mm)	Echo Time (ms)	Repetition Time (ms)	Flip Angle (Degrees)	Inversion Time (ms)
NFBS	SIEMENS MAGNETOM MR B17 (Nathan Kline Institute-Rockland Sample)	T1	1 × 1 × 1	3.02	2600	8	900
MICCAI 2016	Siemens Verio 3T (University Hospital of Rennes)	FL	0.5 × 0.5 × 1.1	400	5000	120	1800
		T1	1 × 1 × 1	2.26	1900	9	NA
	Siemens Aera 1.5T (University Hospital of Lyon)	FL	1.03 × 1.03 × 1.25	336	5000	120	1800
		T1	1.08 × 1.08 × 0.9	3.37	1860	15	NA
	Philips Ingenia 3T (University Hospital of Lyon)	FL	0.74 × 0.74 × 0.7	360	5400	90	1800
		T1	0.74 × 0.74 × 0.85	4.3	9.4	8	NA
IBSI 2015	Philips Medical Systems 3T	FL	0.82 × 0.82 × 2.2	68	NA	NA	835
		T1	0.82 × 0.82 × 1.17	6	10.3	8	NA
HC-FMB	Siemens Verio 3T (Hospital of Clinics—Botucatu Medical School, São Paulo State University	FL	0.43 × 0.43 × 4.6	80	9000	150	2500
		T1	0.47 × 0.47 × 4.6	9	465	69	NA

**Table 2:** Performance values (mean) obtained in the test group with the proposed models for brain extraction and lesion segmentation.

	Brain Segmentation—1st CNN	Lesion Segmentation—2nd CNN
Dice Coefficient	0.9786	0.8893
Accuracy	0.9969	0.9996
Precision	0.9851	0.9376
Sensitivity	0.9851	0.8609
Specificity	0.9985	0.9999

**Table 3:** Comparison of the results obtained for the second CNN with those of other previous methods.

	Dice Coefficient	Precision	Sensitivity	Accuracy	Specificity
Buda et al., 2019 [37]	0.8752	0.9274	0.9030	0.9995	0.9998
Gabr et al., 2020 [14]	0.7839	0.8956	0.7981	0.9994	0.9998
Ghosal et al., 2019 [35]	0.8701	0.9213	0.8911	0.9996	0.9998
Our CNN	**0.8893**	**0.9376**	0.8609	0.9996	**0.9999**

Bold indicates highest values.

## Data Availability

The NFBS repository is available at: http://www.preprocessed-connectomes-project.org/NFB_skullstripped (accessed on 29 November 2021). Access to the MICCAI 2016 dataset is open with access through shanoir (https://shanoir.irisa.fr/shanoir-ng/challenge-request, accessed on 29 November 2021). The IBSI 2015 data set is available at: http://smart-stats-tools.org/lesion-challenge-2015 (accessed on 29 November 2021). The test dataset is not public and the training models and code in the current study are available on reasonable request through the corresponding author’s email (marcela.oliveira@unesp.br).
